# Dengue surveillance using gravid oviposition sticky (GOS) trap and dengue non-structural 1 (NS1) antigen test in Malaysia: randomized controlled trial

**DOI:** 10.1038/s41598-021-04643-4

**Published:** 2022-01-12

**Authors:** Sivaneswari Selvarajoo, Jonathan Wee Kent Liew, Tock H. Chua, Wing Tan, Rafdzah Ahmad Zaki, Romano Ngui, Wan Yusoff Wan Sulaiman, Poo Soon Ong, Indra Vythilingam

**Affiliations:** 1 Department of Parasitology, Faculty of Medicine, Universiti Malaya, 50603 Kuala Lumpur, Malaysia; 2 Department of Pathobiology and Medical Diagnostics, Faculty of Medicine and Health Sciences, Universiti Malaysia Sabah, 88400 Kota Kinabalu, Sabah Malaysia; 3 Department of Social and Preventive Medicine, Faculty of Medicine, Centre for Epidemiology and Evidence Based Practice, Universiti Malaya, 50603 Kuala Lumpur, Malaysia; 4 Petaling Jaya City Council, 46675 Petaling Jaya, Selangor Malaysia; 5 Present Address: Enviromental Health Institute, National Environment Agency, Singapore, 569874 Singapore

**Keywords:** Diseases, Health care

## Abstract

Dengue remains a major public threat and existing dengue control/surveillance programs lack sensitivity and proactivity. More efficient methods are needed. A cluster randomized controlled trial was conducted for 18 months to determine the efficacy of using a combination of gravid oviposition sticky (GOS) traps and dengue non-structural 1 (NS1) antigen for early surveillance of dengue among *Aedes* mosquito. Eight residential apartments were randomly assigned into intervention and control groups. GOS traps were placed at the intervention apartments weekly to trap *Aedes* mosquitoes and these tested for dengue NS1 antigen. When dengue-positive pool was detected, the community were notified and advised to execute protective measures. Fewer dengue cases were recorded in the intervention group than the control. Detection of NS1-positive mosquitoes was significantly associated with GOS *Aedes* index (r_s_ = 0.68, P < 0.01) and occurrence of dengue cases (r_s_ = 0.31, P < 0.01). Participants’ knowledge, attitude, and practice (KAP) toward dengue control indicated significant improvement for knowledge (P < 0.01), practice (P < 0.01) and total scores (P < 0.01). Most respondents thought this surveillance method is good (81.2%) and supported its use nationwide. Thus, GOS trap and dengue NS1 antigen test can supplement the current dengue surveillance/control, in alignment with the advocated integrated vector management for reducing *Aedes*-borne diseases.

## Introduction

Dengue is an important arthropod-borne viral infection that has rapidly spread globally in recent years. About three billion people live in dengue-prone areas with an estimated 100–400 million dengue cases occurring annually on a global scale^1^. An estimated 390 million dengue infections occur per year with only 96 million being clinically apparent^2^, of which more than half the cases are reported in Southeast Asia^3^. In Malaysia, there was a four-fold increase in national dengue cases in the year 2014 due to environmental factors, rapid urbanization, and serotypes switch^4^. Since then, all four serotypes are circulating in Malaysia and dengue disease remains a significant public health threat^5,6^. Female *Aedes aegypti* and *Aedes albopictus* mosquitoes are primary and secondary vectors in Malaysia. The primary vector *Ae. aegypti* is very anthropophilic^7^, day-biting and feeds on several humans during one blood meal if disturbed during feeding^8^. An infected *Ae. aegypti* remains infected for life and can easily cause an epidemic, especially at places where people live in close proximity^9^.

A licensed dengue vaccine, Dengvaxia^10^, has recently become available in the absence of an antiviral cure, but still, it falls short in protection due to its partial efficacy and safety issues^11^. Therefore, vector surveillance remains the critical core of the dengue control program in many Southeast Asian countries including Malaysia^12–14^. This involves source reduction, house to house larval surveys, larviciding and fogging which are reactive and ineffective^15–18^. These are also labour intensive and not cost-effective due to mushrooming of high-rise buildings^19^, lack of community participation and insecticide resistance in the mosquitoes^17,20,21^. Additionally, current vector surveillance/control system have neglected clinically inapparent, dengue-infected individuals who can still spread dengue virus^22,23^.

The World Health Organization (WHO) recently developed Global Vector Control Response (GVCR) to strengthen vector control globally for vector-borne diseases. The critical areas in the routine vector surveillance include identifying the source of dengue transmission, collaborating within the health sector, having an integrated approach including chemical and non-chemical controls, as well as engaging and mobilizing local community for sustainability^1^. Thus, in resource-constrained situations, new dengue surveillance and control paradigm should be economically viable, especially in low-to-middle-income nations where dengue cases are most prevalent.

Our research team had previously published the efficacy of using gravid oviposition sticky (GOS) trap and dengue NS1 antigen rapid test in combination as a new proactive tool for dengue surveillance in Malaysia^24–26^. Briefly, this method uses GOS traps made from simple and readily available materials to trap and test female mosquitoes for the presence of the virus. This method was able to detect dengue-positive mosquitoes one-week before dengue cases were reported^24^. Early detection of dengue-positive mosquitoes allows rapid and prompt actions to be instituted to prevent and mitigate the risks of a dengue epidemic. Dengue surveillance using the reusable and inexpensive GOS traps (US Dollar 0.40 per trap) could be deployed over a large area by a small number of workers. In Singapore, a highly populated and developed country, Gravitrap which is similar to the GOS trap has been used as an *Aedes* monitoring tool since 2013^27^. Moreover, Singapore has also started to use the *Aedes* adult index as a national vector index replacing the larval index^28,29^.

Accordingly, a cluster randomized controlled trial has been conducted to determine the effectiveness of GOS trap and NS1 antigen test kit as an early dengue surveillance/control system. This trial included the active monitoring of *Aedes* adult population using GOS trap, NS1 kit testing, active community engagement and participation in proactive vector control activities as well as both localized and targeted search and destroy.

## Results

### Dengue cases

Throughout the 75 weeks of the trial period, 173 dengue cases and no deaths were reported in the intervention group. On the other hand, a total of 167 including one death and 175 cases were reported in the control and negative control groups respectively. Further analysis by using the GLMM model, indicated no difference between the number of cases recorded between intervention (0.76 ± 0.17) and control groups (0.61 ± 0.16), P = 0.61. However, post hoc test using Tukey method indicated significantly fewer cases were recorded for the individual intervention apartments except for Harmoni, as shown in Table 1.

**Table 1:** Number of dengue cases per week between apartments nested under intervention and control groups (mean ± standard error).

Intervention group		Control group	
Suria	0.09 ± 0.04*	Indah	0.76 ± 0.28*
Park avenue	0.18 ± 0.06*	Vista	1.10 ± 0.32*
Impian	0.22 ± 0.07*	Permai	2.54 ± 0.67
Harmoni	1.36 ± 0.34	Lestari	4.44 ± 1.19

Akaike information Criterion (AIC) = 773.3. Degree of freedom (d.f) = 347.

*Significant difference P value (P < 0.05) in number of cases reported between groups as tested by Tukey’s method.

### Before–after control impact (BACI)

Dengue case data from January 2017 to March 2018 was taken as the “before period” (i.e., prior to GOS deployment) and that from January 2019 to March 2020 as the “after period” (i.e., during GOS deployment) to compare the dengue case ratio. The change in dengue case ratio of the groups before and after GOS trap deployment compared to negative control that was not part of this surveillance study was also investigated (Table 2). The case ratio of intervention group was reduced two times more compared to control group. At the same time, the case ratio in negative control showed an increment of 15.9%. This suggests some evidence of treatment effect after initiation of this proactive surveillance/control in the study area in the year 2020.

**Table 2:** Dengue case ratio in intervention and control groups.

Group	No. of blocks	2017–2018 (case ratio in relation to total dengue cases in the state of Selangor: 29, 908 cases)	2019–2020 (case ratio in relation to total dengue cases in the state of Selangor: 76, 299 cases)	Percentage of change in dengue case ratio (%)
Intervention	35	3.18 × 10^–3^	1.91 × 10^–3^	−39.9
Control	38	2.51 × 10^–3^	2.04 × 10^–3^	−18.7
Negative control	35	2.01 × 10^–3^	2.33 × 10^–3^	15.9

### Temporal analysis

A total of 3751 *Ae. aegypti* (3441 female and 310 male) and 2594 *Ae. albopictus* (2511 female and 83 male) mosquitoes were obtained from the four intervention apartments during 75 weeks of trial. Besides *Ae. aegypti* and *Ae. albopictus*, the GOS traps also captured 3397 *Culex* spp. mosquitoes (2021 female and 1376 male) and *Armigeres* spp. A total of 2319 out of 21,736 traps (10.7%) were considered non-functional. In the control group, a total of 480 *Ae. aegypti* (426 female and 54 male) and 96 *Ae. albopictus* (91 female and 4 male) mosquitoes were obtained from the four control apartments during the 18-month epidemiological survey. Besides *Aedes* mosquito, the GOS traps also captured 56 *Culex* spp. mosquitoes (48 female and 8 male). A total of 525 (24.0% of the 2,186) GOS traps were considered non-functional.

**Figure 1:**
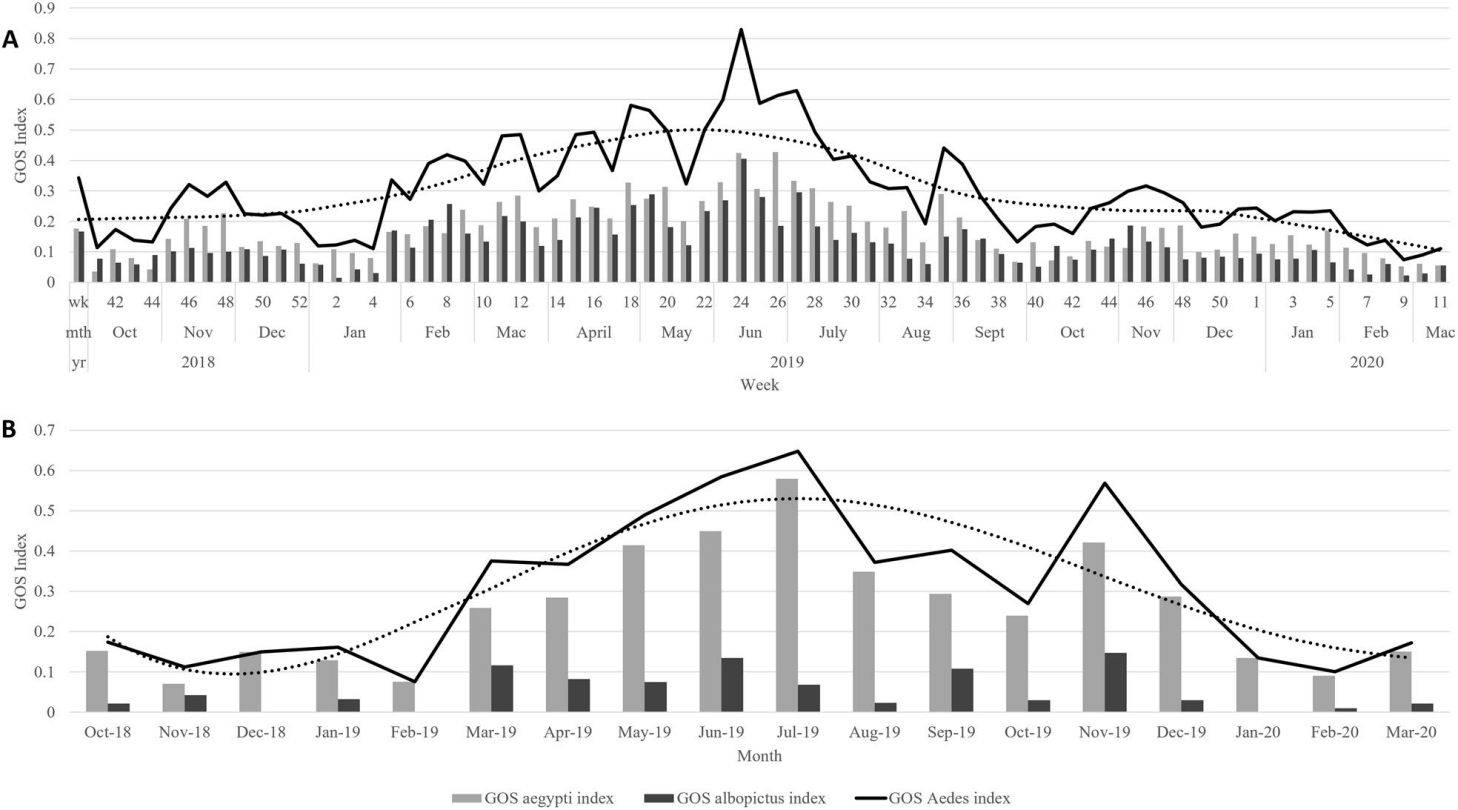
Temporal trend of GOS index weekly in **(A)** Intervention group and monthly, **(B)** control group throughout the study period. The black dotted curve is a trend decomposition of GOS *Aedes* index in the respective group.

The weekly and monthly GOS trap index for *Aedes*, *Ae. aegypti* and *Ae. albopictus* in intervention and control groups are shown in Fig. 1A,B, respectively. In intervention group, the GOS *Aedes* index ranged between 0.07 to 0.83 and averaging at 0.30. Mann–Kendall trend test indicated a significant downward trend (P ≤ 0.04, n = 75, S = −2.01) for GOS *Aedes* index. In addition, as illustrated in Fig. 1A, both *Aedes* spp. populations showed a similar trend and recorded the highest abundance in June 2019 (week 24–26). The mean GOS *Aedes* index decreased by 23.1% when compared between the first six months (0.26) and last six months (0.20) of the trial. This indicated weekly deployment of GOS trap can reduce mosquito abundance.

In control group, the GOS *Aedes* index ranged between 0.07 to 0.64 with an average of 0.30. *Aedes aegypti* was more abundant than *Ae. albopictus* in the control group and a peak abundance was seen in July 2019. Mann–Kendall trend test did not reveal any significant trend (P = 0.68, n = 18, S = 0.42) in GOS *Aedes* index of the control group. In addition, the mean GOS *Aedes* index increased by 52.9% when compared between the first six months (0.17) and last 6 months (0.26) of the trial.

### Spatial analysis

Vertical distribution of *Aedes* mosquitoes was examined between the different blocks. The number of *Aedes* mosquitoes trapped were unevenly distributed among floors in the apartment blocks. As presented in Table 3, in the intervention group, more *Ae. aegypti* were trapped at lower floors (≤ 3 floors) than higher floors (> 4 floors). Whereas in the control group more *Ae. aegypti* appeared at the higher floors (12th and 15th). On the other hand, *Ae. albopictus* were trapped more frequently at lower floors (≤ 3 floors) in both groups. Even among the apartments from similar area, the dominant species at each apartment varied. *Aedes aegypti* was more abundant in Harmoni (Intervention) and Vista (Control) while *Ae. albopictus* in Impian (Intervention) and Indah (Control). Moreover, a high abundance of *Culex* spp. was detected in Suria (Intervention) and Lestari (Control), while other apartments showed mixed breeding of all types of mosquitoes (Supplementary File 1: Table S1). The time series plot for each intervention apartments is displayed in Supplementary File 2: Fig. S1 A–D. Analysis of individual intervention apartments indicated more cases occur in apartments predominated by *Ae. aegypti* (S1 Fig. D). Whereas fewer cases were reported from apartments with predominant *Ae. albopictus* even with the highest GOS *Aedes* index recorded (S1 Fig. A).

**Table 3:** Number of *Ae. aegypti* caught per trap per week in each floor in intervention and control groups (mean ± standard error).

Intervention group (df = 5719)		Control group (df = 1180)	
Ground	0.95 ± 0.08*	Ground	0.69 ± 0.12
F1	1.11 ± 0.35*	F3	0.48 ± 0.09
F2	1.13 ± 0.32*	F6	0.13 ± 0.10
F3	0.56 ± 0.05	F9	0.33 ± 0.18
F5	0.33 ± 0.10	F12	3.38 ± 1.48*
F8	0.34 ± 0.10	F15	0.98 ± 0.59*
F11	0.21 ± 0.07		
F14	0.29 ± 0.09		
F17	0.36 ± 0.11		

*Significant difference P value (P < 0.05) in number of *Ae. aegypti* per trap per week within group.

In addition, the distribution of dengue cases by floors collectively for both study groups were examined (Supplementary File 2 Fig. 2). Overall, more dengue cases were reported from lower floors of apartment blocks regardless of groups. However, by proportion, the dengue cases from lower floors were predominantly contributed by the intervention group whereas those from higher floors by the control group. Correspondingly, this may be due to the higher *Ae. aegypti* density in respective floors of the intervention and control groups.

**Figure 2:**
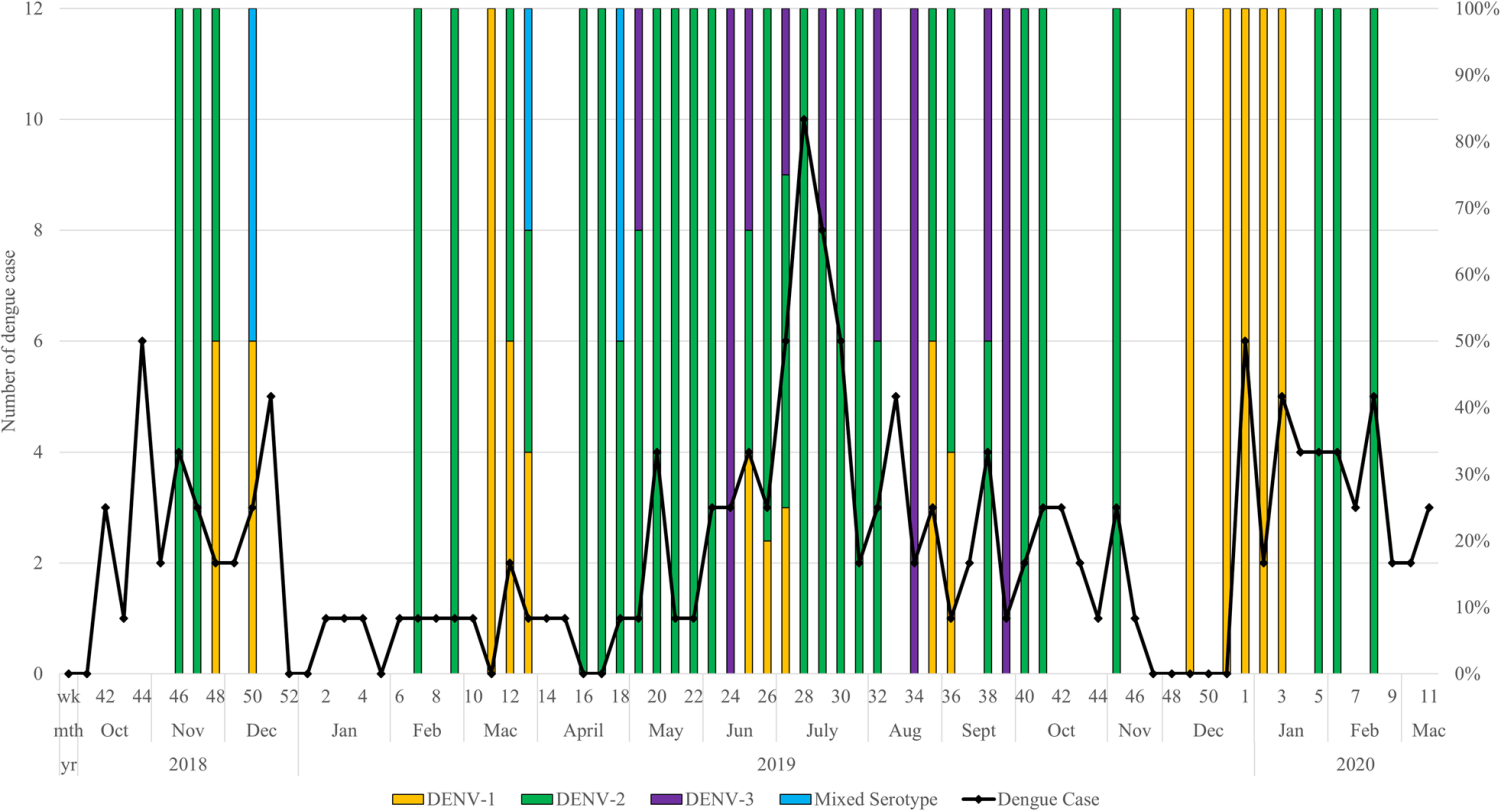
Distribution of dengue serotypes in mosquito sample and dengue cases by week.

### Dengue-positive *Aedes* mosquito detected in the intervention group

Throughout this study, 71 out of 1020 pools (6.96%) of *Ae. aegypti* abdomen and 6 out of 676 (0.89%) pools of *Ae. albopictus* abdomens from the intervention group were positive by NS1 antigen test.

The dengue virus serotypes detected in the abdomen pools were as follows: dengue virus serotype 1, DENV-1:15; DENV-2: 44; DENV-3: 9; DENV-2 + DENV-3: 1; DENV-2 + DENV-4: 2). Figure 2 shows the distribution of dengue serotypes in the mosquitoes and dengue cases weekly. The DENV-2 (61.9%) serotype was prevalent throughout the study period followed by DENV-1 (21.1%). A surge in DENV-3 (12.6%) serotype was recorded between May-Sept 2019. Similarly, dengue cases were seen to increase during this period.

### Relationship of entomological finding and dengue cases

**Figure 3:**
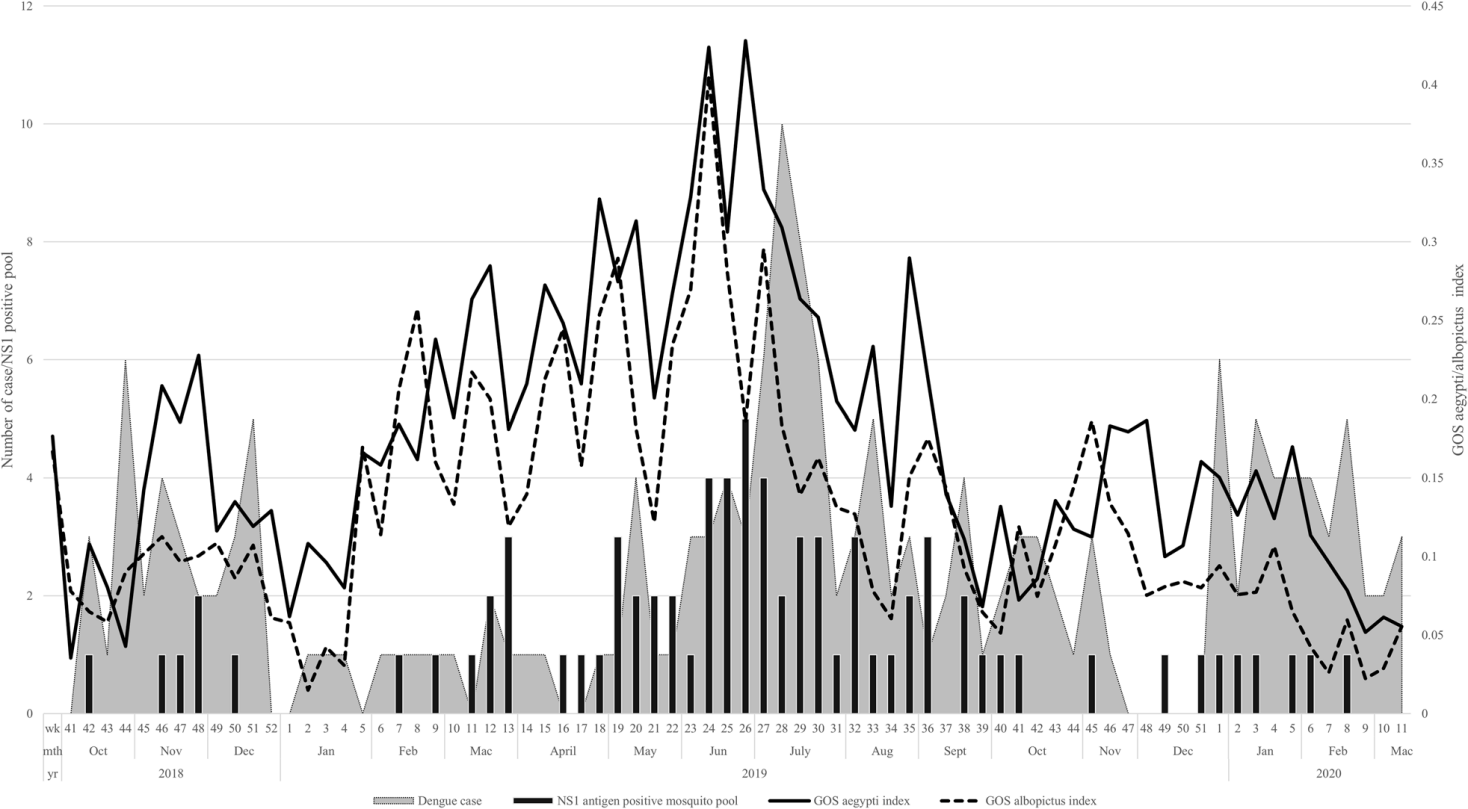
Weekly GOS index, the onset of dengue cases and dengue NS1 positive pool throughout the study period in the intervention group.

Seventy-seven pools of dengue NS1 antigen positive mosquitoes were detected from the intervention group. The relationships of the number of dengue cases with the number of NS1 antigen positive pools and GOS index are depicted in Fig. 3. The first dengue-positive mosquito was seen in the same week when cases were reported. As illustrated in Fig. 3, a peak in dengue cases in July (week 28–29) occurred at lag of two weeks after the peak in the number of NS1 antigen positive pools in June (week 24–26).

There was a significant positive relationship between NS1 antigen positive mosquito pool with dengue case (r_s_ = 0.38, P < 0.01). This indicates that the presence of NS1 positive mosquitoes implies an increased risk of transmission and occurrence of dengue cases. Moreover, increase in GOS *Aedes* index (r_s_ = 0.68, P < 0.01), GOS *aegypti* index (r_s_ = 0.70, P < 0.01) and GOS *albopictus* index (r_s_ = 0.54, P < 0.01) was significantly related to detection of dengue NS1 antigen positive mosquito pool. However, there was no correlation between GOS *Aedes* index (r_s_ = 0.18, P = 0.12), GOS *aegypti* index (r_s_ = 0.12, P = 0.12) and GOS *albopictus* index (r_s_ = 0.04, P = 0.76) with dengue cases which indicate dengue cases are not affected by mosquito abundances.

### Occurrence of asymptomatic dengue in the intervention group

A total 407 volunteers were tested for the presence of dengue virus following detection of dengue positive mosquitoes in proximity to their residences in 41 weeks of sampling out of the 75 weeks. The residents were approached with the approval of management bodies in the same week upon detection of dengue-positive mosquito pools. Among those tested, 14 (3.43%) were positive by NS1 antigen rapid test kit on the spot. Upon one month follow-up of these individuals, 6 (1.47%) individuals were confirmed dengue-asymptomatic because they were healthy and did not have any sign/symptoms of dengue whereas 8 (1.97%) were pre-symptomatic. Real time PCR performed at the end of the of the trial retrospectively revealed 34 (8.35%) residents were actually positive for dengue. These individuals did not show any sign and symptom of dengue during sampling time. Therefore, the most notable finding was uncovering individuals infected with the dengue virus upon detection of dengue-positive mosquitoes. Part of the sampling result has been published in Tan, et al. (2020).

### Knowledge, attitude, and practice of the study popular

In the intervention group, 224 residents (86 males and 138 females) participated in the pre-KAP assessment (Supplementary Table S2), and 229 residents (94 male and 135 female) participated in the post-KAP assessment study (Supplementary Table S3). There were more female participants (60.3%) than males, and Malays (69.0%) were the predominant race. Mann–Whitney U-test revealed a significant increase in the participant’s knowledge (P < 0.01), practice (P < 0.01), and total scores (P < 0.01), but not for attitude (P = 0.22) after the trial. Based on an 80% cut-off value, the number of participants who had good knowledge (Supplementary Table S4), attitude (Supplementary Table S5) and practice (Supplementary Table S6) towards dengue prevention increased from 49.6% (n = 111), 50.9% (n = 115), 41.2% (n = 93) to 58.5% (n = 134), 51.1% (n = 117), 58.5% (n = 134) respectively. Additionally, with regards to the post-intervention scores, there was a significant positive correlation between attitude and practice (r_s_ = 0.18, P < 0.01).

### Residents feedback

Most respondents opined that the surveillance method used in this study was either good (39.5%) or very good (41.7%) and supported its use nationwide (Supplementary Table S7). 65–80% agreed that the said surveillance method effectively reduced *Aedes* mosquito density and dengue case counts, apart from providing an early warning on the presence of dengue-positive mosquitoes. On the other hand, 8 respondents felt uncomfortable having health authorities visiting their premises.

## Discussion

In Malaysia, dengue control and surveillance mechanism have not changed since 1970s. Routine house-to-house *Aedes* larval survey followed by source reduction and chemical control when cases were reported remains mainstay dengue control measure in Malaysia and most Southeast Asian countries^14,17,30^. Although it has been reported that these methods lack effectiveness in the current scenario, they are still being used^12,18,31^. Therefore, this randomized control trial is to establish an integrated dengue surveillance incorporating entomological, virological, and clinical systems to detect early cases, followed by immediate and targeted prevention and control measures.

First and foremost, the intervention has contributed to dengue case control. BACI analysis revealed a 39.9% in dengue case ratio in the intervention group compared to 18.7% reduction in dengue case ratio in the control group. On the other hand, dengue case ratio increased in the negative control group which received only the standard vector control measures by MOH. This reflects a positive outcome of this proactive surveillance/control, where actions are taken immediately after detection of dengue-positive mosquitoes or a rise in mosquito density. Similarly, in Singapore, the deployment of Gravitraps contributed to a 36% reduction in dengue case burden in the intervention sites when compared to the control sites^29^. In comparison, the rise in dengue cases seen in the negative control group may indicate the inadequacy of the current reactive surveillance/control by the health authorities. Although, the GLMM model indicated no difference between the number of cases recorded between intervention and control groups, this was mainly due to the higher number of cases in one of the intervention apartments while significantly lower number of cases was recorded for the other three intervention apartments. This apartment was the only apartment that reported multiple outbreaks throughout the study period. This may be due to an outbreak ongoing in this apartment even before the trial started. Moreover, it can be linked to population density and mobilization effects, as that one apartment is surrounded by shops and consists of 19 blocks where high population movement occurs. The movement of viraemic individuals in and out of this area may be infecting the mosquitoes^32^. A dense population has also been postulated as one of the factors causing increased dengue virus transmission^33,34^. Likewise, in our earlier KAP and seroprevalence study, there was a significant association between dengue IgG positivity and type of residential buildings^35^.

This surveillance method also removes infected mosquitoes that drive dengue transmissions without the use of insecticide. Comparison of GOS *Aedes* index at the beginning and at the end of the trial revealed a reduction in mosquito abundance in the intervention group. In contrast, fogging and ULV using pyrethroid which were carried out when cases were reported in the control apartments, did not affect the total number of mosquitoes caught. A continuous increase in GOS *Aedes* index can been seen in apartments in the control group. Although the insecticide resistance status of mosquito population in the study was not investigated, insecticide resistance is highly prevalent especially in dengue hotspot areas in Selangor^20,36^.

It should be noted that the GOS trap is not the sole intervention of this trial. The positive outcomes of this trial can be attributed to the early public alert of communities and health authorities followed by the early preventive actions taken, which were all triggered by proactive surveillance using the GOS trap. The surveillance/control method employed has promoted active participation of the community and encouraged them to be more responsible in dengue prevention and control. Thus, upon detection of a dengue-positive mosquitoes in housing units especially high-rise apartments, more targeted actions such as house inspection, notification, intense education, or search destroy can be taken as an alternative intervention.

Engaging the community and educating the public in vector surveillance/control are vital in ensuring in-built sustainability. The GOS traps are very cheap and can be easily made. Surveillance using the GOS traps can be carried out by the community with help from the health authorities. As evidence, our previous study in another site had successfully implemented *Aedes*/dengue surveillance using GOS traps inside residential units and dengue-positive mosquitoes can be detected within one hour in the field without laboratory requirements. Moreover, vector/dengue management and control upon detection of dengue-positive mosquitoes were thoroughly conducted by the COMBI team and Public Health Units with limited input from researchers^26^.

Additionally, this surveillance/control method is useful in examining the spatial–temporal patterns of the *Aedes* mosquitoes. In this study, *Ae. aegypti* is most abundant at the lower floors of the intervention group and at higher floors of the control group. Correspondingly, the apartments and floors with high number of *Ae. aegypti* caught, reported a higher number of dengue cases as well. It can be postulated that the predominance of *Ae. aegypti* may lead to a higher dengue transmission risk since it can be a more efficient vector of dengue than *Ae. albopictus*^37,38^. Previous studies have indicated *Ae. aegypti* prefers to oviposit near ground level^39^ and seldom disperses beyond 100 m after a meal^8,40^. Hence, spatial heterogeneity of the *Ae. aegypti* population among sites, is a possible contributing factor to the differential transmissions and occurrences of dengue cases. Thus, regular entomological surveillance using simple and cheap adult trap should be considered in vector surveillance/control.

The result of this randomized control trial further supports the notion that dengue surveillance using GOS trap and dengue NS1 test can detect dengue-positive mosquitoes before dengue cases were reported^24,25^. Trapping dengue NS1 positive mosquito was found to be related to GOS *Aedes* index and dengue cases, while there was no correlation between GOS index and dengue cases. In Singapore, treatment site with higher Gravitrap *aegypti* index found to be associated with higher mean number of dengue cases per week^29^. However, other studies have reported that the *Aedes* index does not always reflect dengue transmission^41,42^. Moreover, the significant positive correlation of dengue cases with the number of NS1 positive mosquito pool shows detection of dengue positive mosquito can be used as an indicator of viral transmission. This is similar to findings in Thailand where only infected *Aedes* were positively associated with dengue cases^43^. Thus, NS1 positive mosquitoes and monitoring adult mosquito index as carried out in this study, should be a good indicator for initiation of vector control instead of dengue case. Detection of dengue NS1 antigen using abdomen or whole mosquito provides reliable results because NS1 antigen tests are rapid and the NS1 antigen is stable, while viral RNA may degrade over time. The finding of dengue NS1-positive abdomens but dengue negative heads/thoraces could mean that the dengue virus in the mosquito was still in its incubation period after a blood meal.

This proactive surveillance further introduces a paradigm of a targeted and early detection of dengue serotype shift using dengue NS1 positive mosquitoes. In Malaysia, the serotype distribution has been inconsistent and switching of DENV serotype is a significant cause of outbreak^4,44^. In this study, DENV-2 followed by DENV-1 were the most predominant serotype in the mosquito samples. However interestingly, a surge in the DENV-3 serotype was detected in the dengue NS1 positive mosquitoes during mid-year which corresponded to high occurrence of dengue cases in humans. It has been documented that the introduction of DENV-3 was often associated with outbreaks of severe dengue in other countries like Brazil^45^, China^46^ and Indonesia^47^. Thus, virological surveillance in NS1 positive mosquito samples can serve as an epidemic alert system and hence continuous monitoring should be considered.

On another note, the detection of asymptomatic viraemic individuals in this trial clearly showed the presence of asymptomatic cases, which have otherwise been largely ignored in dengue surveillance and control. Yet there is a lack of information on the epidemiology of asymptomatic dengue. It was highly likely that asymptomatic dengue plays a role in the maintenance of dengue transmission in the absence of an epidemic and may account for 84% of all dengue transmissions^48^. In Thailand, of the 5.7% rate of total virus infection school students, 87% of these were either asymptomatic or mild^49^. Symptomatic secondary dengue infection and inapparent dengue may also have nearly equal incidence among elementary students^50^. Even though the prevalence of inapparent dengue varies by geographical location, time, and demography^51–53^, it has been reported that such individuals (which include asymptomatic, pre-symptomatic, and sub-clinical cases) can still transmit dengue to mosquitoes^22^. In Malaysia, the seroprevalence of dengue ranges from 28 to 94% (depending on the subset of population being studied), and over 90% of individuals will invariably have a history of dengue seropositivity as early as the age of 45^54–56^. In previous studies such individuals were detected from a mass clinical study^22^ or retrospective studies^57,58^, which made it challenging to predict dengue transmissions among asymptomatic individuals. To the best of our knowledge, this is the first study that demonstrates a dengue surveillance system which is able to capture asymptomatic, dengue-viraemic individuals living in an area with infected mosquitoes. Duong, et al. (2015) have demonstrated that apparently healthy individuals without any signs and symptoms were more infectious to *Aedes* mosquitoes. This could mean that the existing, reactive actions taken to control dengue transmission after the initial reports of dengue cases, are delayed and hence likely to be, futile as the disease would have been widespread by then^23^. Infected *Aedes* mosquitoes can serve as an early warning and identification of such mosquitoes should be the way forward for proactive dengue surveillance and control measures.

The sustainable implementation of any dengue surveillance/control method requires cooperation and participation from the community. Therefore, it is essential that the community understand and adopts positive attitudes towards dengue monitoring and control effects. Our earlier findings before the trial started^35^ are similar to results of previous studies showing Malaysian communities generally have good knowledge of dengue and a positive attitude on dengue prevention, but these are not translated to good practices of dengue prevention^59–61^. Post-trial intervention revealed a significant increase in the number of residents having good practices towards dengue prevention. This could have been due to our active engagement with the community by providing warnings of an impending outbreak and prompting them to be more vigilant and to keep their residences free of breeding sites. Active health campaign in collaboration with COMBI team and notification of dengue-positive mosquitoes through social media, posters and banners actively kept them aware of the dengue situation within their housing units. However, respondents did not show a significant increase in attitude scores after the trial. A similar observation was obtained from KAP studies^62,63^ when assessing attitudes-beliefs change after conducting dengue educational board game. This could be rationalized by the fact that multiple interventions over more extended periods are needed to change attitude and beliefs relating to health.

This randomized control trial provides a proactive and targeted surveillance not only to prevent and control dengue disease, but also Zika and chikungunya viruses if they are present in the mosquitoes. However, several problems have been encountered such as missing traps, trap tampering, and the unwillingness of residents to have traps placed directly beside their premises during initial stage of the trial. Nevertheless, some of these issues were overcome by educating and explaining the importance to the residents and towards the end, participating residents were more welcoming of this approach. Another limitation of the study was its relatively short duration of one and a half years. Arguably, surveillance over a more extended period of two to three years would provide better and more robust evidence. On another note, this randomised control trial has employed architecturally different apartments as units of item of randomisation instead of individual blocks. Nonetheless, the advantages of the surveillance method described here lie in its simplicity and demonstrated efficacy. As mentioned, 81.2% of the community felt that this surveillance method was suitable and supported its use nationwide.

Thus, this proactive paradigm makes it an integrated vector management system and can be implemented in a bottom-up fashion. As a result, the GOS trap and dengue NS1 antigen test, may be used as a dengue surveillance tool to augment the existing monitoring and control system, in line with the advocated integrated vector management approach for reducing *Aedes*-borne diseases. This approach is useful in high-rise urban areas where people live close to each other, and few infected mosquitoes can transmit dengue to many people.

## Methodology

### Study design and participants

**Figure 4:**
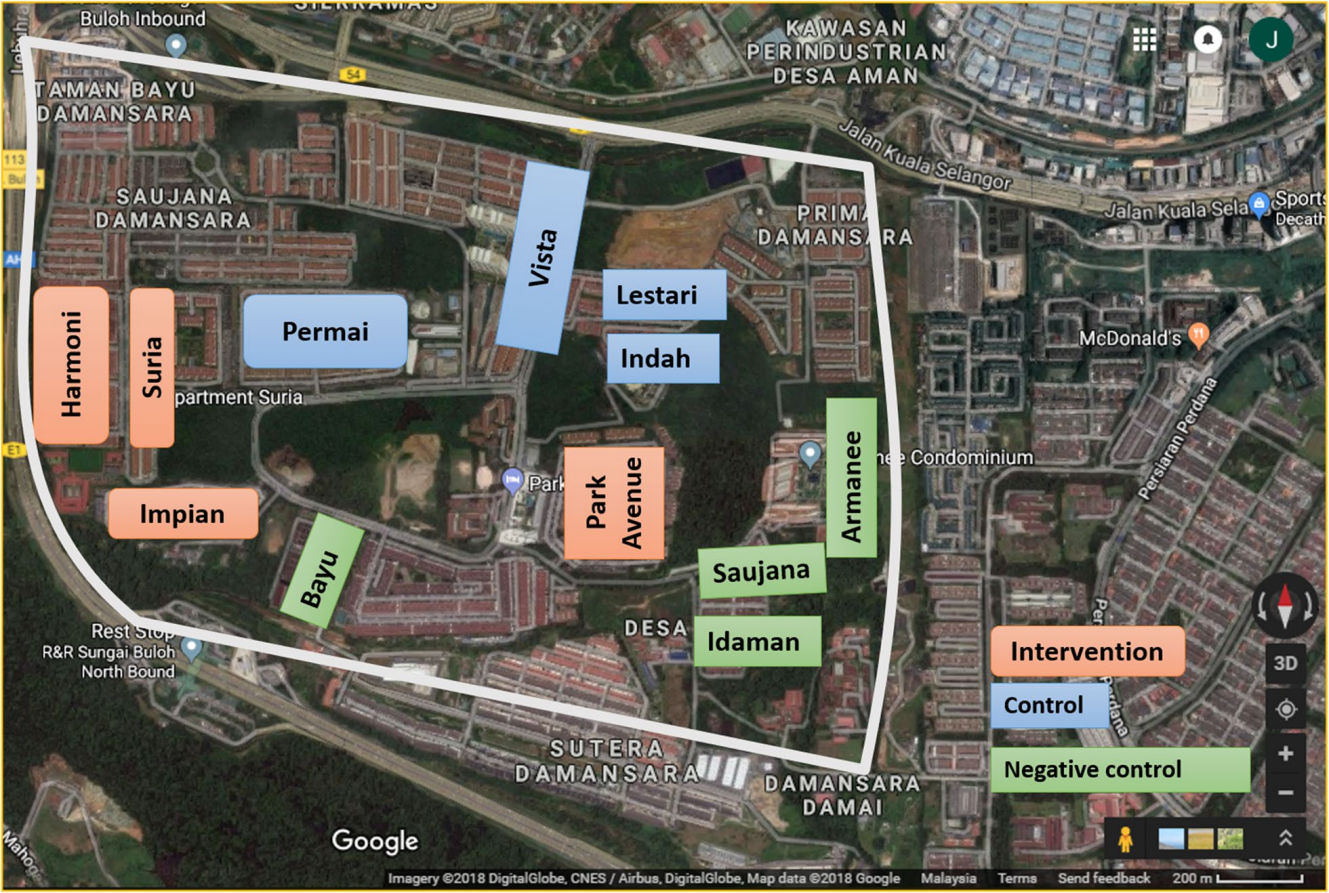
Map of study area at Damansara Damai. “Map data @2018 Google”.

This community-based, parallel-cluster randomized controlled trial study was conducted at eight apartments of Damansara Damai or Petaling Jaya Utara 10 (3.1930°N, 101.5923°E), Petaling district, Selangor, Malaysia for 18 months from October 2018 to March 2020 (Fig. 4). This interventional trial was registered at ClinicalTrials.gov (ID: NCT03799237), on January 2019 retrospectively.

### Ethics approval and consent to participate

The research protocol was approved by the Medical Research Ethics Committee, University Malaya Medical Center, Malaysia (MRECID.No: 2018525-6321). All residents/residential units of eight sampled were eligible for this study. Informed consent was obtained from all the study participants. The study adhered to the principles of biomedical research involving human subjects. Specifically, the rights, integrity, and confidentiality of the research subjects, in addition to the credibility and accuracy of the reported results, will be protected as per the Declaration of Helsinki. Written informed consent was obtained from participants prior to the administration of the KAP questionnaire and/or performance of venipunctures by the trained researchers. The participants were not reimbursed in any form.

### Study procedure

**Figure 5:**
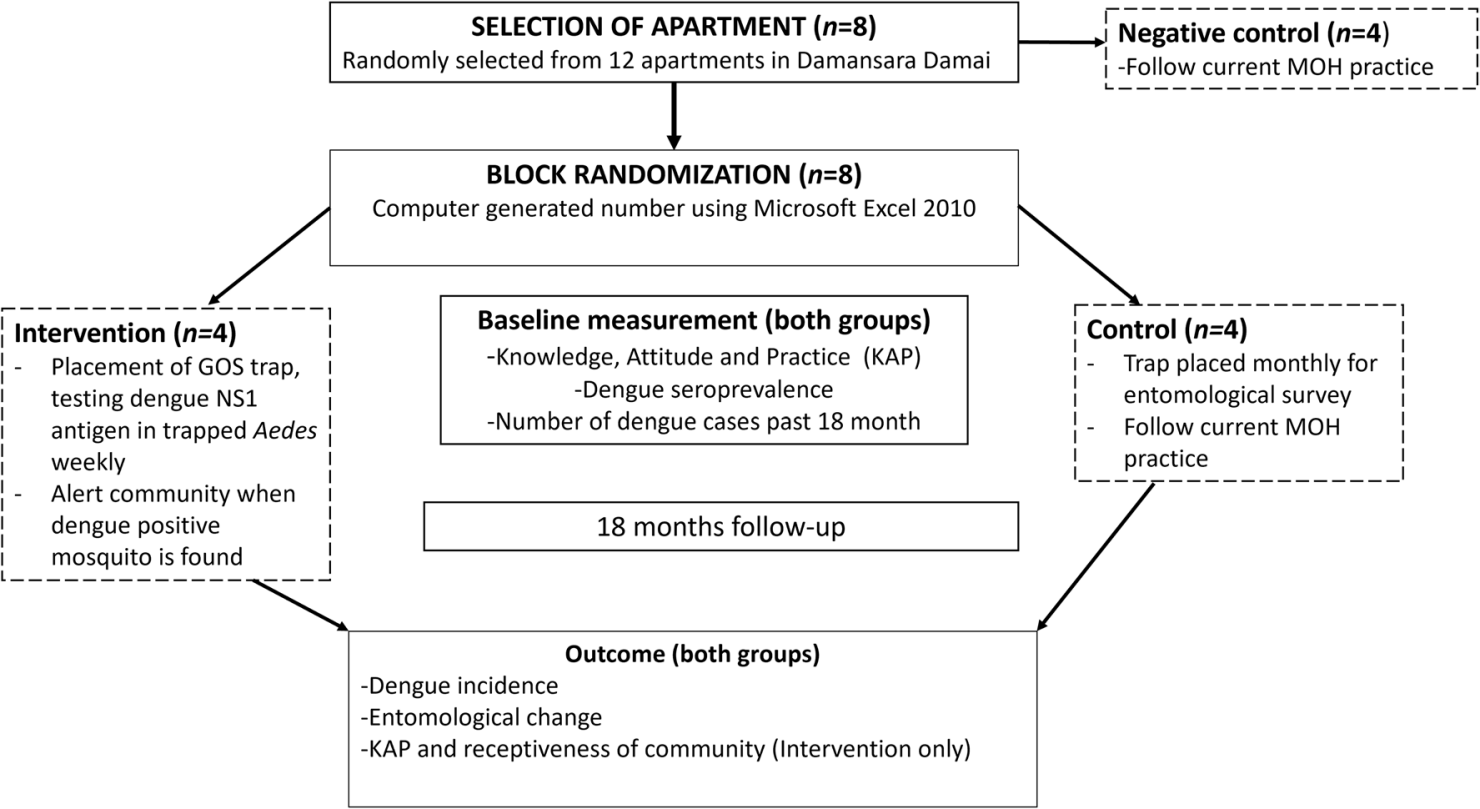
Flowchart of the study trial.

The study protocol has been published previously^64^. Figure 5 shows the flowchart of the study trial. This trial was conducted at Damansara Damai, Petaling Jaya Utara 10, PJU10 (3.1930°N, 101.5923°E) –Petaling District, Selangor, Malaysia. It were chosen because it is "closed" area (there is only one main entrance and exit to this area), it has sufficient number of high-rise apartments and it being a dengue hotspot. In brief, eight residential apartments were selected and four each were randomly assigned into intervention and control groups. In the intervention group, GOS traps were placed and checked weekly. Female *Aedes* mosquitoes trapped were tested for the presence of dengue NS1 antigen using the SD Biosensor Standard Q Dengue NS1 test kit (Gyeonggi-Do, South Korea) and was serotyped using RT-PCR according to previously published protocols^23,55^. When the NS1 test was positive, flyers and posters were disseminated within two days to notify the affected apartment's management and residents that dengue-positive mosquito(es) had been detected. The Vector Control Unit of the Petaling Jaya City Council was also notified. The flyers/posters presented information of (1) the apartment block(s) where the dengue-positive mosquito(es) was found, as well as (2) preventive measures for residents to protect themselves from mosquito bites. The apartment’s management also used social media such as WhatsApp or Facebook as a medium to disseminate the said information to the residents. Concurrently, mosquito repellents (Fumakilla Nobite Lotion, active ingredient: 10% icaridin; and NATMOS anti-mosquito spray, active ingredient: lemongrass and lemon eucalyptus oil) and flyers were distributed door-to-door, as well as posters displayed at strategic locations such as the notice boards of each block. The residents were briefly reminded to take necessary precautionary measures against dengue and perform search-and-destroy activities. In addition, upon detection of NS1 positive mosquitoes people residing within a radius of 50 m from the trap were requested to undergo finger-prick NS1 antigen test door-to door to detect occurrences of asymptomatic dengue^23^. The samplings were performed in tandem with the intervention of the trial, which is during notification of dengue positive mosquitoes in the neighbourhood and education on dengue prevention. Upon written consent, details like age, any recent signs and symptoms, history of dengue infection and family history of dengue infection were taken. Approximately 3 drops of finger-pricked blood or 100 μL of venous blood were collected and used directly on the SD Biosensor Standard Q Dengue NS1 test kit (Gyeonggi-Do, South Korea). Three blood spots were made on a Whatman Filter Paper. As per the manufacturer's instructions, results were interpreted within 15 to 20 min. Individuals positive for dengue NS1 antigen were advised to take precautionary measures to protect themselves and others from mosquito bites by applying repellent, and to seek medical attention if symptoms occurred. Dengue NS1-positive individuals without any signs and symptoms were followed up for 4 weeks to determine further development of any signs and symptoms of dengue. Occasionally, the research team also organized health campaign and search and destroy activities in collaboration with the management bodies and Communication for Behavioural Impact (COMBI) team when continuous increase in GOS *Aedes* index was detected in the apartment. On the other hand, a reduced number of GOS traps were placed in the control group, once a month for entomological survey only^55^. In addition, the negative control group was not part of the surveillance/deployment study and is only used for epidemiological comparisons. All apartments in this study were subjected to regular and normal vector control measures by Ministry of Health. The measures consisted of pyrethroid-fogging at the apartments when new dengue cases were reported. Larval surveys were also conducted depending on the severity of the outbreaks and the availability of manpower^4^.

In addition, assessment on knowledge, attitude, and practice (KAP) toward dengue prevention was conducted before and after the trial in the intervention group using a self-administered questionnaire. Additional questions about their feedback and receptiveness on the new method of dengue surveillance were also asked in the post-KAP survey. The result of the KAP assessment in all eight participating apartments before the trial started has already been published^35^.

### Entomological data

Entomological indices for GOS trap were calculated as follow,

$$\text{GOS }Aedes\text{ index}=\frac{\text{total number of female adult }Aedes\, \text{mosquitoes}}{\text{number of functional GOS traps}}$$

$$\text{GOS }aegypti\text{ index}=\frac{\text{total number of female adult }Ae. aegypti}{\text{number of functional GOS traps}}$$

$$\text{GOS }albopictus\text{ index}=\frac{\text{total number of female adult }Ae. albopictus}{\text{number of functional GOS traps}}$$

The GOS index calculation was adapted from Ong, et al. (2020) and previously used in Liew, et al. (2021), whereby the total number of adult female *Aedes*, *Ae. aegypti* or *Ae. albopictus* populations were normalized with number of functional GOS trap collected weekly. Functional GOS traps refer to fully assembled GOS traps where the inner-lining brown paper with sticky glue remained in the small container. Traps that were empty, destroyed, missing, without inner-lining brown paper were considered non-functional.

### Dengue cases data

The weekly dengue cases data were obtained from the District Health office. Under the Prevention and Control of Infectious Diseases Act, it is mandatory for all private and public health facilities to notify all clinically diagnosed and laboratory confirmed dengue cases within 24 h of diagnosis to the nearest health office^6^. The residential address and onset date of each dengue case were obtained and anonymized prior to analysis.

### Statistical analysis

Entomological data analyses were performed using R programming language version 3.5.2 (R Foundation for Statistical Computing, Vienna, Austria) and MS Excel 2019 program. Two-tailed Mann–Kendall test was conducted to identify the presence of trends in the weekly and monthly GOS *Aedes* index in intervention and control groups, using the rank-test function. One-tailed Mann Kendall test was then used to further analyse if the determined trends were of increasing or decreasing trend. Generalized linear mixed models (GLMM using glmmTMB package) were constructed to analyse the number of mosquitoes caught per week per trap, number of dengue NS1 positive mosquitoes and dengue incidence; and differences in mosquitoes caught at different apartments, blocks, and floors. The GLMM model testing was done for *Ae*. *aegypti* and *Ae*. *albopictus*.

In GLMM testing, the frequency of sampling (whether weekly or monthly) was considered a fixed factor (Intervention). The different apartments having the same sampling frequency were nested under each group. Sampling occasions was used as a random factor. As the number of traps that were recovered varied each week, offset for traps was used. Poisson distribution and zero inflation were also incorporated in the analysis. The trap data for different apartments, blocks and floors were also subjected to GLMM to determine if there was any difference between the number of mosquitoes trapped. This was done separately for weekly and monthly trapping frequency for different floors. Models testing associations between response variables (e.g., mosquito abundance) and explanatory variables (e.g., frequency of sampling, blocks, or floors) were assessed through model comparison based whichever with a higher log-likelihood and lower Akaike information criterion (AIC) values. Tukey post hoc contrasts were used to differentiate the nature of statistical differences between different levels of the explanatory variables.

A before-after control impact (BACI) statistical design was used to examine the effect of the intervention on dengue transmission^29^ to compensate for temporal and seasonal differences between the intervention and control groups. The change in the dengue case ratio of the study site before and after trap deployment was calculated. In this analysis, the intervention and control groups were also compared against the negative control group where no GOS trap surveillance/deployment was conducted.

For KAP component, data analysis was performed using the Statistical Package for Social Science version 23 (SPSS, Inc., Chicago, IL). A scoring system was utilized to evaluate the KAP data as published earlier^35^. Moreover, changes in KAP levels among residents were analysed using the unpaired t-test.

## Supplementary Information

Supplementary Tables.

Supplementary Figure S1.

Supplementary Figure S2.

## Data Availability

The dataset of this study is available upon request from the corresponding author.
