# Supplementary material for: Dengue surveillance using gravid oviposition sticky (GOS) trap and dengue non-structural 1 (NS1) antigen test in Malaysia: randomized controlled trial
Source: Sci Rep. 2022 Jan 12;12:571. doi: 10.1038/s41598-021-04643-4 (PMC8755775; doi:10.1038/s41598-021-04643-4)
Supplement: Supplementary file 1 — Supplementary Tables. [file 41598_2021_4643_MOESM1_ESM.docx]

| **Group** | **Type of mosquito** | **Apartments** | **No/trap/week *** | **AIC** | **df** |
| --- | --- | --- | --- | --- | --- |
| **Intervention** | *Ae. aegypti* | Harmoni | 1.029±0.0668 * | 11592.2 | 5725 |
|  |  | Impian | 0.663±0.0571 |  |  |
|  |  | Park Avenue | 0.502±0.0444 |  |  |
|  |  | Suria | 0.743±0.0455 |  |  |
|  | *Ae. albopictus* | Harmoni | 0.198±0.0187 | 8476.9 |  |
|  |  | Impian | 2.190±0.1536 * |  |  |
|  |  | Park Avenue | 0.216±0.0270 |  |  |
|  |  | Suria | 0.765±0.0541 |  |  |
|  | *Culex* spp. | Harmoni | 0.039±0.0081 | 5465.4 |  |
|  |  | Impian | 0.035±0.0084 |  |  |
|  |  | Park Avenue | 0.031±0.0075 |  |  |
|  |  | Suria | 0.373±0.0689 * |  |  |
| **Control** | *Ae. aegypti* | Indah | 0.107±0.1107 | 1774.3 | 1182 |
|  |  | Lestari | 0.470±0.1023 |  |  |
|  |  | Permai | 0.392±0.0888 |  |  |
|  |  | Vista | 0.782±0.1355 * |  |  |
|  | *Ae. albopictus* | Indah | 0.7850±0.4829 * | 558.2 |  |
|  |  | Lestari | 0.0729±0.0443 |  |  |
|  |  | Permai | 0.1584±0.0732 |  |  |
|  |  | Vista | 0.0823±0.0372 |  |  |
|  | *Culex* spp. | Indah | 0.1396±0.1669 | 410.1 |  |
|  |  | Lestari | 0.1641±0.1044 |  |  |
|  |  | Permai | 0.0814±0.0547 |  |  |
|  |  | Vista | 0.1110±0.0647 |  |  |

**Table S1.** Number of mosquitoes caught per trap per week at different apartments. The GLMM analysis was performed separately for different groups (mean ± standard error). AIC,Akaike information Criterion. Degree of freedom (d.f) =347. *Significant difference (P<0.05) in number of mosquito caught per trap per week within group.

| **Characteristic** | | **n (%)** |
| --- | --- | --- |
| Gender (n=224) | Male | 86 (38.4) |
|  | Female | 138 (61.6) |
| Age group, (n=209) | ≤35 | 104 (49.8) |
|  | >35 | 105 (50.2) |
| Race (n=224) | Malay | 138 (61.6) |
|  | Chinese | 17 (7.6) |
|  | Indian | 55 (24.5) |
|  | Others | 14 (6.3) |
| Marital status (n=223) | Single | 54 (24.2) |
|  | Married | 168 (75.3) |
|  | Widowed | 1 (0.4) |
| Education (n=221) | None | 3 (1.5) |
|  | Primary | 16 (7.2) |
|  | Secondary | 125(56.5) |
|  | Tertiary | 77 (34.8) |
| Income (n=215) | < MYR 1,000 | 122 (56.8) |
|  | MYR 1,001 – 3,000 | 56 (26.0) |
|  | >MYR 3,001 | 37 (17.2) |
| History of dengue (n=220) | Yes | 36 (16.4) |
|  | No | 184 (83.6) |

**Table S2.** Sociodemographic characteristics of study population during pre KAP assesement.

| **Characteristic** | | **n (%)** |
| --- | --- | --- |
| Gender (n=229) | Male | 94 (41.0) |
|  | Female | 135 (59.0) |
| Age group (n=214) | ≤35 | 73 (34.1) |
|  | >35 | 141 (65.9) |
| Race (n=229) | Malay | 176 (76.9) |
|  | Chinese | 4 (1.7) |
|  | Indian | 30 (13.1) |
|  | Others | 19 (8.3) |
| Marital status (n=229) | Single | 49 (21.4) |
|  | Married | 161 (70.3) |
|  | Widowed | 19 (8.3) |
| Education (n=226) | None | 3 (1.3) |
|  | Primary | 9 (3.9) |
|  | Secondary | 146 (64.8) |
|  | Tertiary | 68 (30.0) |
| Income (n=211) | < MYR 1,000 | 63 (29.9) |
|  | MYR 1,001 – 3,000 | 109 (51.6) |
|  | >MYR 3,001 | 39 (18.5) |
| History of dengue (n=229) | Yes | 41 (17.9) |
|  | No | 188 (82.1) |

**Table S3.** Sociodemographic characteristics of study population during post KAP assesement.

| **Statement** | | **Correct knowledge, n (%)** | |
| --- | --- | --- | --- |
|  |  | **Pre** | **Post** |
| Dengue virus caused by virus | | 175 (82.2) | 179(78.2) |
| Dengue transmitted by mosquito bite | | 217 (96.4) | 224(97.8) |
| *Aedes Aegypti* and *Aedes albopictus* are main vector for dengue in Malaysia | | 187 (89.0) | 225 (98.2) |
| *Aedes* mosquito prefer to breed in clean water | | 154 (70.0) | 140 (61.1) |
| *Aedes* mosquito can breed both indoor and outdoor location | | 199 (91.3) | 219 (95.2) |
| Clean water as little 5 milliliter enough for mosquito to breed | | 183 (88.0) | 195 (85.2) |
| Life cycle (Eggs-Larvae-pupae-adult) | | 199 (93.9) | 202 (88.2) |
| 10 complete days required for maturation of mosquito | | 33 (16.3) | 139 (60.7) |
| Egg can survive in dry condition up to 6 months | | 103 (49.8) | 86 (37.6) |
| All fish cannot be used to kill *Aedes* mosquito | | 105 (51.2) | 103 (45.0) |
| Mosquitoes like to bite early morning and late evening | | 193 (91.5) | 203 (89.8) |
| Dengue fever can cause death | | 198 (92.5) | 225 (98.3) |
| You and your family member are at risk of dengue | | 127 (58.5) | 181 (80.1) |
| Dengue can infect same person more than once | | 135 (62.2) | 194 (85.8) |
| Sign of dengue | High grade fever | 203 (94.4) | 223 (97.4) |
|  | Headache | 199 (93.4) | 222 (96.9) |
|  | Muscle pain | 194 (91.1) | 216 (94.3) |
|  | Joint pain | 188 (89.1) | 221 (96.5) |
|  | Rashes | 180 (84.1) | 221(96.5) |
|  | Pain behind the eyeball | 159 (75.0) | 215 (93.9) |
|  | Persistent vomiting or diarrhoea | 183 (85.5) | 172 (76.1) |
|  | Rapid breathing | 141 (67.1) | 203 (88.6) |
|  | Severe abdominal pain | 151 (72.2) | 175 (76.4) |
|  | Bleeding from nose or gums | 153 (72.5) | 193 (84.3) |
|  | Restlessness | 144 (69.4) | 169 (73.8) |

**Table S4.** Knowledge about dengue in pre and post KAP assesement of study population.

| **Variable** | | **Pre, n(%)** | **Post, n(%)** |
| --- | --- | --- | --- |
| I want to help to reduce the number of dengue cases in my area | Yes | 190 (89.6) | 205 (89.5) |
|  | No | 8 (3.8) | 5 (2.2) |
|  | Not sure | 14 (6.6) | 19 (8.3) |
| I check dengue situation or hotspots around my area regularly | Yes | 149 (70.3) | 150 (65.5) |
|  | No | 63 (29.7) | 79 (34.5) |
| I will take extra action to prevent dengue infection if I know the risk of being infected with dengue is increasing in my area | Yes | 177 (85.1) | 190 (84.1) |
|  | No | 8 (3.8) | 11 (4.8) |
|  | Not sure | 23 (11.1) | 25 (11.1) |
| Removal of mosquito breeding sites at my premises will reduce the chance of dengue infection among my family members | Yes | 189 (90.9) | 206 (90.4) |
|  | No | 6 (2.9) | 8 (3.5) |
|  | Not sure | 13 (6.3) | 14 (6.1) |
| Chemical fogging by health authority is good enough to prevent dengue infection | Yes | 102 (48.6) | 69 (30.1) |
|  | No | 70 (33.3) | 125 (54.6) |
|  | Not sure | 38 (18.1) | 35 (15.3) |
| It is necessary to continue the removal of mosquito breeding sites at home even during the period when there’s no outbreak | Yes | 191 (91.8) | 203 (88.6) |
|  | No | 9 (4.3) | 4 (1.7) |
|  | Not sure | 8 (3.8) | 22 (9.6) |
| Dengue outbreak in my community can be controlled if every household is committed to remove mosquito breeding sites | Yes | 189 (91.3) | 215 (93.8) |
|  | No | 7 (3.4) | 5 (2.2) |
|  | Not sure | 11 (5.3) | 9 (3.9) |
| I will take part in a public activity for dengue control or removal of mosquito breeding sites | Yes | 161 (77.4) | 185 (80.8) |
|  | No | 8 (3.8) | 8 (3.5) |
|  | Not sure | 39 (18.8) | 36 (15.7) |

**Table S5**. Attitude towads dengue preventation in pre and post KAP assesement.

| **Variable** | | **Pre, n(%)** | | **Post, n(%)** |
| --- | --- | --- | --- | --- |
| Calling health authority for fogging (n=102) | Yes | 200 (93.9) | | 212 (93.0) |
|  | No | 13 (6.1) | | 16 (7.0) |
| Calling private pest control (n=100) | Yes | 125 (64.0) | | 138 (60.3) |
|  | No | 82 (39.6) | | 90 (39.3) |
| Search and destroy breeding site (n=100) | Yes | 203 (95.3) | | 223 (97.4) |
|  | No | 10 (4.7) | | 6 (2.6) |
| Use mosquito spray (n=102) | Yes | 196 (94.2) | | 217 (94.8) |
|  | No | 12 (5.8) | | 12 (5.2) |
| Which best describes Search and Destroy? (n=100) | Discard water with larvae | 32 (15.4) | | 27 (11.8) |
|  | Discard water with larvae and wash container with antiseptic | 51 (24.5) | | 29 (24.5) |
|  | Discard stagnant water and scrub the container | 111 (53.4) | | 155 (67.7) |
|  | Discard stagnant water and wash container with hot water | 14 (6.7) | | 18 (7.9) |
| Best self-prevention method (n=98) | Mosquito repellent | Yes | 128 (60.7) | 141 (61.6) |
|  |  | No | 26 (26.5) | 88 (38.4) |
|  | Bed nets | Yes | 108 (51.2) | 136 (59.4) |
|  |  | No | 28 (28.6) | 93 (40.6) |
|  | Remove breeding sites | Yes | 166 (78.7) | 207 (90.4) |
|  |  | No | 12 (12.2) | 22 (9.6) |
|  | Insecticide | Yes | 132 (62.6) | 153 (66.8) |
|  |  | No | 78 (37.1) | 76 (33.2) |

**Table S6.** Dengue prevention practice of the respondents in pre and post KAP assesment

| After this study, do you know more about the methods of mosquito/dengue control used by the health authorities? (n=225) | | Yes | 201 (89.3) |
| --- | --- | --- | --- |
|  |  | No | 5 (2.2) |
|  |  | No difference | 19 (8.4) |
| What is your opinion on the *Aedes*/dengue surveillance method (GOS trap & NS1 kit) used in this study? (n=228) | | Very good | 95 (41.7) |
|  |  | Good | 90 (39.5) |
|  |  | Unfavourable | 5 (2.2) |
|  |  | Very unfavourable | 5 (2.2) |
|  |  | Not sure | 33 (14.5) |
| Will you support this surveillance method (GOS trap & NS1 kit) to be used nationwide? (n=228) | | Yes | 185 (81.1) |
|  |  | No | 10 (4.4) |
|  |  | Not sure | 33 (14.5) |
| Yes, I support this surveillance method (GOS trap & NS1 kit) to be used nationwide? (n=179) | This method could reduce the number of mosquitoes in my housing area | | 161 (89.9) |
|  | This method could reduce the number of dengue cases | | 148 (82.7) |
|  | This method alerts me of dengue infested mosquito in my housing area, so I take precaution | | 121 (67.6) |
|  | This method appears to be more efficient than the current methods used by the health authorities. | | 117 (65.4) |
| No, I do not support this surveillance method (GOS trap & NS1 kit) to be used nationwide? (n=8) | This method not effective at reducing the number of mosquitoes in my housing area | | 1 |
|  | This method is not effective at reducing the number of dengue cases | | 2 |
|  | I feel uncomfortable having the health authorities come to my premise for examination/blood test | | 3 |
|  | This method does not prevent dengue cases from happening | | 2 |

**Table S7.** Residents’ feedback
