# Supplementary material for: Dengue surveillance using gravid oviposition sticky (GOS) trap and dengue non-structural 1 (NS1) antigen test in Malaysia: randomized controlled trial
Source: Sci Rep. 2022 Jan 12;12:571. doi: 10.1038/s41598-021-04643-4 (PMC8755775; doi:10.1038/s41598-021-04643-4)
Supplement: Supplementary file 2 — Supplementary Figure S1. [file 41598_2021_4643_MOESM2_ESM.pdf]

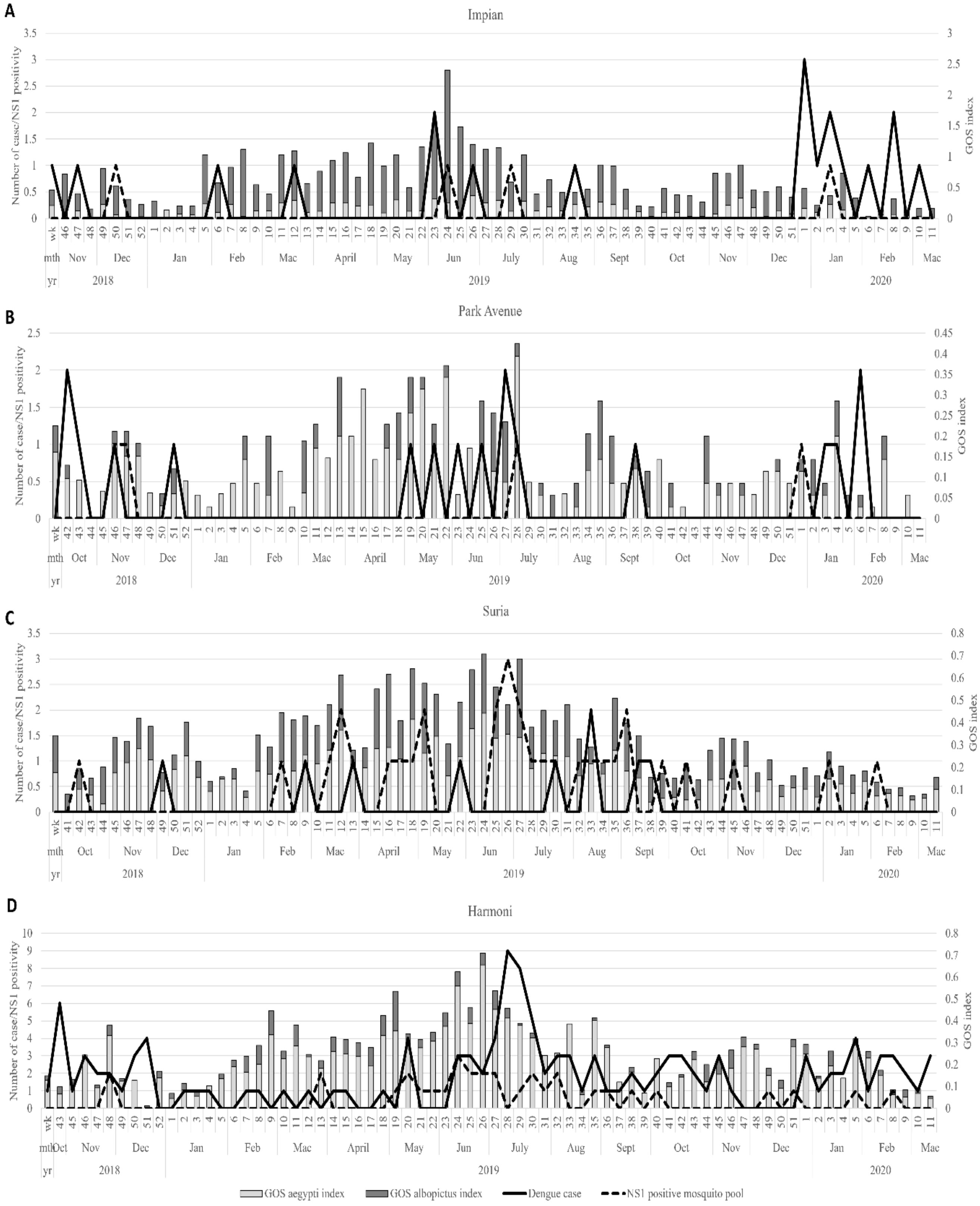

Supplementary file 2: Fig S1 A-D. Temporal trend of the weekly GOS index, dengue case and NS1 positive pool of each intervention apartment.
