# Supplementary material for: Dengue surveillance using gravid oviposition sticky (GOS) trap and dengue non-structural 1 (NS1) antigen test in Malaysia: randomized controlled trial
Source: Sci Rep. 2022 Jan 12;12:571. doi: 10.1038/s41598-021-04643-4 (PMC8755775; doi:10.1038/s41598-021-04643-4)
Supplement: Supplementary file 3 — Supplementary Figure S2. [file 41598_2021_4643_MOESM3_ESM.pdf]

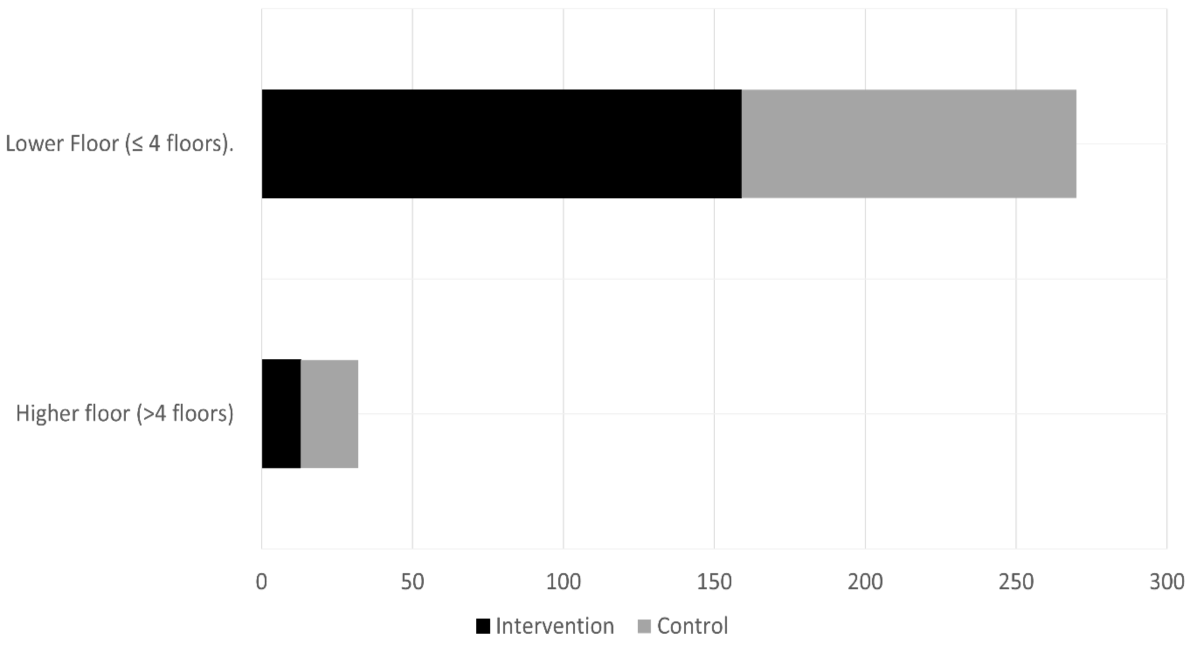

Supplementary file 3: Fig S2. A total number of dengue cases reported by aggregated floor in intervention and control group throughout study period.
